# Use of contrast enhanced magnetic resonance angiography in assessment of anatomic suitability for renal denervation in the hypertensive population

**DOI:** 10.1186/1532-429X-16-S1-P147

**Published:** 2014-01-16

**Authors:** Claire E Raphael, Aamir Ali, Vassilis Vassiliou, Hitesh Patel, Arun J Baksi, Sanjay K Prasad, Carlo Di Mario, Dudley J Pennell, Raad Mohiaddin

**Affiliations:** 1 Department of CMR, Royal Brompton Hospital, London, UK; 2 Department of Cardiology, Royal Brompton Hospital, London, UK

## Background

Renal denervation (RDN) is an effective treatment for resistant hypertension with expanding indications in the hypertensive population [1]. The European Society of Hypertension (ESH) guidelines [2], largely based on the Symplicity trial inclusion criteria, state RDN should not be performed if the patient has multiple renal arteries, renal artery stenosis or renal arteries with a diameter of less than 4 mm or length of less than 20 mm. Imaging prior to consideration of RDN is recommended but has not been included in all RDN trial protocols. The proportion of hypertensive patients with anatomy suitable for RDN using the current guidelines is not known.

## Methods

A series of 112 consecutive contrast enhanced magnetic resonance angiograms performed at our institution for investigation of hypertension over a period of one year (September 2012-2013) were retrospectively analysed for anatomic suitability for RDN. Images were acquired using a dedicated 1.5T scanner with an eight-channel phased-array receiver coil (Siemens Magnetom Avanto). Multiplanar reconstruction and raw images were assessed for the presence of accessory renal arteries and renal artery stenosis. Renal artery dimensions were measured by two operators blinded to clinical results.

## Results

The population was 60% male with a mean age of 48 ± 17 years. 38% (n = 42) of patients had multiple renal arteries, including 5% with bilateral dual renal artery supply. 5% (6 patients) had significant renal artery stenosis. Agreement between operators for vessel dimensions was good (weighted kappa 0.6). The mean main renal artery calibre was 6.3 ± 3.5 mm. 25 patients (22%) had at least one main renal artery with a diameter less than 4 mm. While accessory renal arteries were smaller than main renal arteries, with a mean diameter of 3.3 ± 1.1 mm, 28% had a diameter of 4 mm or greater. One patient (0.8%) had a renal artery length less than 20 mm. Overall, 30% of patients did not meet the current anatomic criteria for RDN.

## Conclusions

Under the current ESH guidelines, 30% of the hypertensive patients studied had anatomy unsuitable for RDN under the current guidelines, most commonly due to the presence of accessory renal arteries. Our data support the use of non-invasive renal angiography prior to consideration of RDN and may prompt further studies of safety and efficacy in populations with anatomy outside of current guidelines. While accessory arteries were usually small, a significant proportion (28%) were of sufficient calibre for RDN and in these cases, an approach targeting accessory as well as main renal arteries may be suitable.

## Funding

This project was supported by the NIHR Cardiovascular Biomedical Research Unit of Royal Brompton and Harefield NHS Foundation Trust.
